# Understanding Oral Lichen Planus and Its Malignant Potential in the Saudi Arabian Population: A Systematic Review

**DOI:** 10.3290/j.ohpd.c_2337

**Published:** 2025-11-20

**Authors:** Ali Alqarni, Shaimaa M. Alarabi, Muhannad A. Alamri, Abdullah A. Alzamil, Ibrahim A. Alhebshi, Murayziq A. Algethami, Khalid Aljohani, / Khlood A. Alkurdi, Abdullah F. Alshammari

**Affiliations:** a Ali Alqarni Assistant Professor, Department of Oral and Maxillofacial Surgery and Diagnostic Sciences, Faculty of Dentistry, Taif University, Taif, Saudi Arabia. Research concept, study design, supervision, statistical analysis, wrote the original draft, evaluated and approved the final manuscript.; b Shaimaa M. Alarabi Assistant Professor and Consultant, Department of Oral and Maxillofacial Surgery and Diagnostic Sciences, Faculty of Dentistry, Taif University, Taif, Saudi Arabia. Research concept, study design, supervision, statistical analysis, wrote the original draft, evaluated and approved the final manuscript.; c Muhannad A. Alamri General Dentist, Faculty of Dentistry, Taif University, Taif, Saudi Arabia. Data collection, wrote the original draft, evaluated and approved the final manuscript.; d Abdullah A. Alzamil General Dentist, Faculty of Dentistry, Taif University, Taif, Saudi Arabia. Data collection, wrote the original draft, evaluated and approved the final manuscript.; e Ibrahim A. Alhebshi General Dentist, Faculty of Dentistry, Taif University, Taif, Saudi Arabia. Data collection, wrote the original draft, evaluated and approved the final manuscript.; f Murayziq A. Algethami Periodontist, Faculty of Dentistry, Taif University, Taif, Saudi Arabia. Data collection, wrote the original draft, evaluated and approved the final manuscript.; g Khalid Aljohani Associate Professor, Department of Diagnostic Oral Sciences, Faculty of Dentistry, King Abdulaziz University, Jeddah, Saudi Arabia. Research concept, study design, supervision, statistical analysis, wrote the original draft, evaluated and approved the final manuscript.; h Khlood A. Alkurdi Senior Registrar, Ministry of Health, Qassim Health Cluster, King Saud Hospital, Unayzah, Kingdom of Saudi Arabia. Data collection, wrote the original draft, evaluated and approved the final manuscript.; i Abdullah F. Alshammari Assistant Professor and Consultant, Department of Basic Dental and Medical Science, College of Dentistry, University of Ha’il, Ha’il, Saudi Arabia. Research concept, study design, supervision, statistical analysis, wrote the original draft, evaluated and approved the final manuscript.

**Keywords:** comorbitiy, malignant transformation, oral lichen planus, oral potentially malignant disorder, prevalence, Saudi Arabia.

## Abstract

**Purpose:**

Oral lichen planus (OLP) is a chronic inflammatory disorder recognised as a potentially malignant disorder of the oral cavity. This review aimed to synthesize available evidence from Saudi Arabia regarding OLP prevalence, clinical presentation, associated factors, and risk of malignant transformation.

**Materials and Methods:**

A systematic search was conducted across several electronic databases (e.g., PubMed, Scopus, Saudi Digital Library) up to February 2024. Eligible studies were original clinical investigations on OLP in Saudi Arabia, reporting at least one of the following: prevalence, clinical presentation, treatment, or malignant transformation. Study quality was appraised using the Joanna Briggs Institute (JBI) checklists.

**Results:**

Eleven studies — eight cross-sectional and three case–control — met the inclusion criteria. Reported prevalence of OLP across Saudi Arabian populations ranged from 0.35% to 11.08%. Clinical forms most often included the reticular variant, followed by erythematous and erosive forms. Topical corticosteroids were the most frequently applied treatment. Reported associations included diabetes mellitus, hypertension, thyroid disease, and hepatitis C virus infection. Histopathological confirmation was not consistently applied across studies. Dysplastic changes were noted in up to 12% of cases, while malignant transformation rates ranged from 0.2% to 5.6%.

**Conclusion:**

Prevalence estimates of OLP in Saudi Arabia are highly variable, reflecting methodological and diagnostic inconsistencies. There is a possible relationship with systemic comorbidities, particularly hepatitis C infection. Multi-center, prospective studies with standardized diagnostic and follow-up protocols are needed to accurately determine epidemiology and malignant potential.

Oral lichen planus (OLP) is a chronic T-cell mediated inflammatory disease that predominantly affects mucous membranes, with the oral cavity being the most frequently involved site.^17,38^ First described by Wilson in 1868,^45^ OLP presents clinically as reticular, erosive, or atrophic lesions, often causing significant pain and impacting quality of life.^16,20,33,34^ Unlike its cutaneous counterpart, which is typically self-limiting, OLP persists chronically in most patients and has been classified by the World Health Organization as an oral potentially malignant disorder (OPMD) with a transformation risk ranging from 0.4% to 5.6%.^44^

The pathogenesis of OLP remains incompletely understood but involves a complex interplay of genetic, immunological, and environmental factors.^19,20,21,25,29,30^ Notably, associations with hepatitis C virus (HCV) infection have been reported globally, though with significant geographical variation.^4,31^ In Saudi Arabia, where HCV prevalence is estimated at 5.6%,^4^ this potential link warrants particular attention given its implications for screening and management.

Despite numerous epidemiological studies of OLP worldwide,^24,25,34,44^ data from Saudi Arabia remain limited and heterogeneous.^2,3,5,6,7,10,12,13,14,22,26,32,39,40,42^ Reported prevalence rates in Saudi Arabia vary dramatically from 0.35% to 11.08%,^3, 5,6,10,13,14,22,26,32,40,42^ likely reflecting differences in diagnostic criteria, study populations, and regional factors.^3,5,10,13,14,22, 32,40,42^ This variability underscores the need for a comprehensive synthesis of existing evidence to clarify disease patterns and guide clinical practice.

The current systematic review addresses three critical gaps:

The lack of standardized data on OLP prevalence and risk factors specific to Saudi populations
Uncertainties regarding the HCV-OLP association in the Saudi context
Limited documentation of management approaches and outcomes in regional clinical practice.

By systematically evaluating available evidence, this study aims to provide clinicians and researchers with a clearer understanding of OLP’s epidemiology, clinical characteristics, and malignant potential in Saudi Arabia. The findings will inform both clinical decision-making and future research priorities in the region.

## MATERIALS AND METHODS

A rigorous systematic approach was employed to identify and synthesize evidence on oral lichen planus (OLP) in the Saudi population. The review protocol was registered prospectively with PROSPERO (registration number: CRD42024567890) and adhered to PRISMA guidelines.^35^

### Search Strategy and Data Sources

A comprehensive literature search was conducted across multiple electronic databases, including PubMed, Scopus, Web of Science, Cochrane Library, Saudi Digital Library, BMC Oral Health, and others. The search encompassed articles published up to February 2024 and utilized key terms related to OLP and Saudi Arabia, including “oral lichen planus,” “lichen planus,” “oral mucosal lesions,” and “Saudi Arabia.” Search restrictions included language (English and Arabic) and study type (clinical studies).

### Study Selection and Eligibility Criteria

Studies were selected based on predefined inclusion and exclusion criteria. Eligible studies were original clinical investigations reporting on:

Prevalence, epidemiology, or incidence of OLP in Saudi Arabia
Clinical and histopathological characteristics
Risk factors, including systemic comorbidities
Malignant transformation or cancer risk.

**Fig 1:**
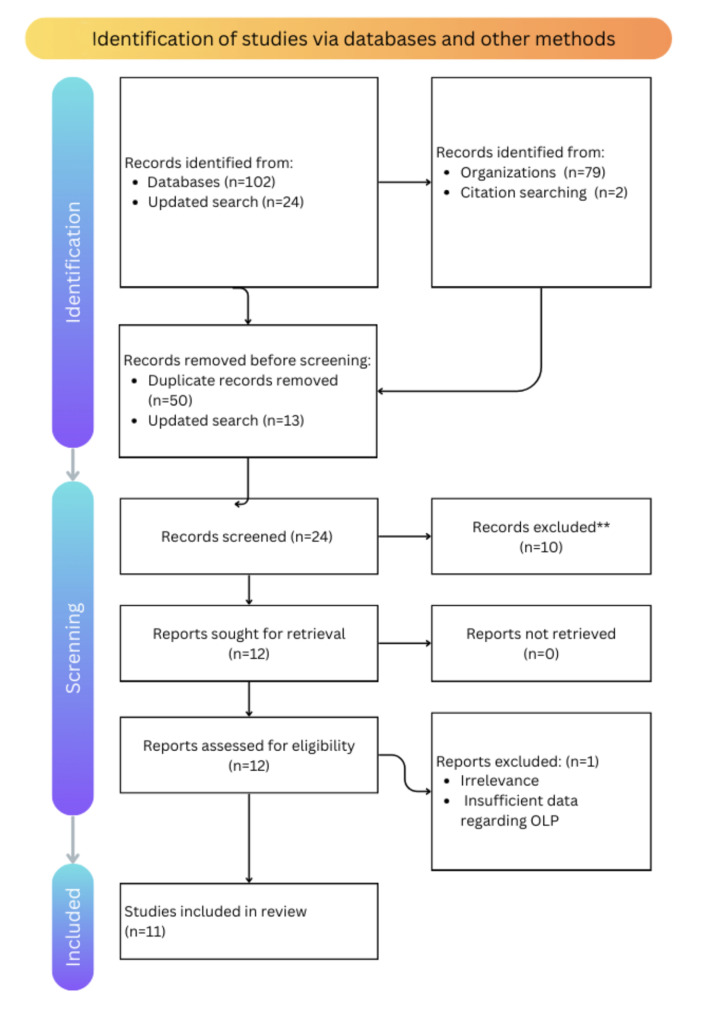
The PRISMA flowchart showing the selection process of the articles retrieved from different online databases.

Exclusion criteria encompassed case reports, reviews, and studies with fewer than 10 patients. Two independent reviewers conducted the study screening and selection, resolving disagreements by consensus or consultation with a third reviewer. Figure 1 illustrates the screening process.

### Data Extraction and Calibration

Data extraction was performed independently by two reviewers using a standardized data extraction form. Extracted items included study design, location, patient demographics, diagnostic criteria, clinical presentation, treatment modalities, and outcomes related to malignant transformation. Prior to full extraction, reviewers calibrated their approach by independently extracting data from a subset of studies to ensure consistency (Cohen’s Kappa: 0.89).

### Quality Assessment and Risk of Bias

**Fig 2:**
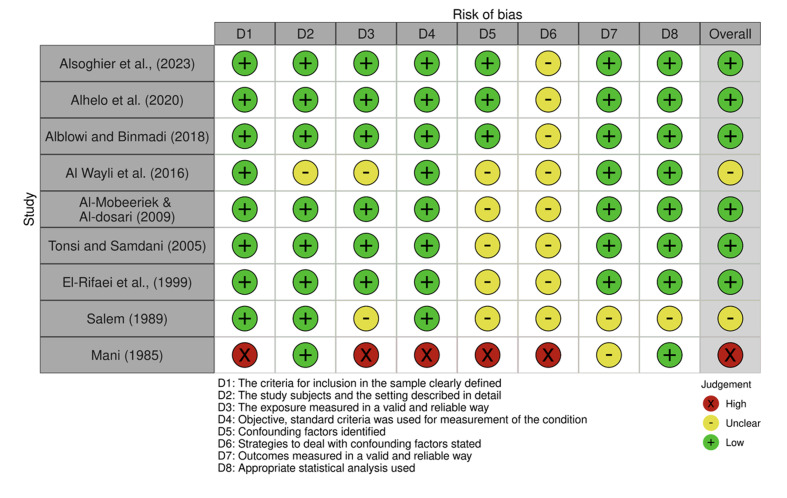
Traffic-light plot of the risk of bias assessment for cross-sectional studies.

**Fig 3:**
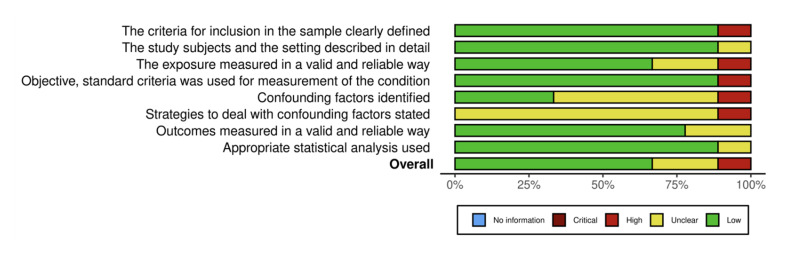
Summary plot for risk of bias assessment results for cross- sectional studies.

**Fig 4:**
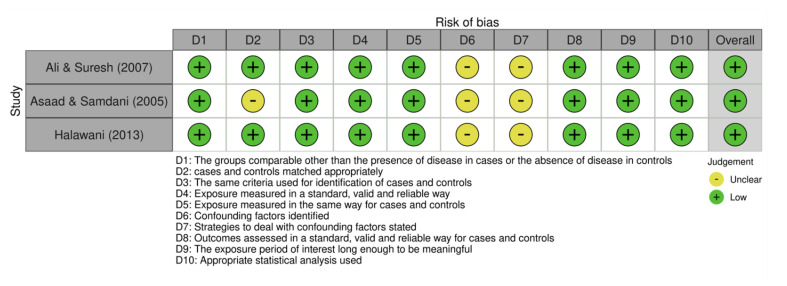
Traffic-light plot of the risk of bias assessment for case control studies.

**Fig 5:**
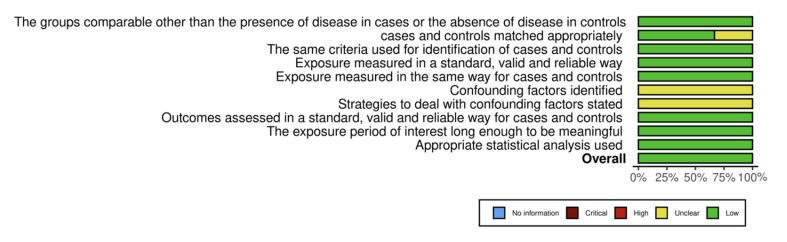
Summary plot for risk of bias assessment results for case control studies.

Each included study’s methodological quality was appraised using the Joanna Briggs Institute (JBI) checklists tailored for cross-sectional and case-control studies.^27^ Scoring assigned 1 point for “Yes,” 0 for “No,” and 0.5 for “Unclear.” Quality scores for included studies ranged from 2.5 to 7.7 for cross-sectional designs and 9 or 10 for case-control studies. Detailed results of quality assessment are presented in Tables 1 and 2. Risk of bias was also depicted graphically through traffic-light plots to visually highlight areas of concern, notably confounding factors (Figs 2 to 5).

**Table 1:** Quality assessment results for crosssectional studies (JBI checklist*)

Author (year)	Q1	Q2	Q3	Q4	Q5	Q6	Q7	Q8	Total (/8)
Alsoghier et al (2023)	Yes	Yes	Yes	Yes	Yes	Unclear	Yes	Yes	7.5
Alhelo et al (2020)	Yes	Yes	Yes	Yes	Yes	Unclear	Yes	Yes	7.5
Alblowi and Binmadi (2018)	Yes	Yes	Yes	Yes	Yes	Unclear	Yes	Yes	7.5
Al Wayli et al (2016)	Yes	Unclear	Unclear	Yes	Unclear	Unclear	Yes	Yes	6.0
AlMobeeriek and Aldosari (2009)	Yes	Yes	Yes	Yes	Unclear	Unclear	Yes	Yes	7.0
Tonsi and Samdani (2005)	Yes	Yes	Yes	Yes	Unclear	Unclear	Yes	Yes	7.0
ElRifaei et al (1999)	Yes	Yes	Yes	Yes	Unclear	Unclear	Yes	Yes	7.0
Salem (1989)	Yes	Yes	Unclear	Yes	Unclear	Unclear	Unclear	Unclear	5.5
Mani (1985)	No	Yes	No	No	No	No	Unclear	Yes	2.5
*JBI crosssectional study criteria: Q1: Inclusion criteria defined; Q2: Subjects and setting described; Q3: Exposure measured validly; Q4: Objective criteria used; Q5: Confounding factors identified; Q6: Confounders addressed; Q7: Outcomes measured validly; Q8: Appropriate statistical analysis.

**Table 2:** Quality assessment results for case–control studies (JBI checklist*)

Author (year)	Q1	Q2	Q3	Q4	Q5	Q6	Q7	Q8	Q9	Q10	Total (/10)
Ali and Suresh (2007)	Yes	Yes	Yes	Yes	Yes	Unclear	Unclear	Yes	Yes	Yes	9.0
Halawani (2013)	Yes	Yes	Yes	Yes	Yes	Unclear	Unclear	Yes	Yes	Yes	9.0
*JBI case–control study criteria: Q1: Groups comparable; Q2: Appropriate matching; Q3: Same criteria for cases/controls; Q4: Exposure measured validly; Q5: Same method for cases and controls; Q6: Confounders identified; Q7: Confounders addressed; Q8: Outcomes assessed validly; Q9: Exposure period adequate; Q10: Appropriate statistical analysis.

### Data Synthesis

Due to heterogeneity in study designs, diagnostic methodologies, and reported outcomes, a narrative synthesis approach was adopted. Where possible, subgroup analyses were performed based on diagnostic criteria (clinical vs histopathological), geographic region within Saudi Arabia, and study period (pre- and post-year 2000). Summarized clinical and epidemiological data were tabulated for comparison (Table 4).

**Table 4:** Summary of main findings from included studies

Author year	n patients	Mean age in years ± SD	F:M ratio	Prevalence OLP	Systemic disease comorbidities	Clinical types	Sites involved	Treatments	Improvement (%)	Malignant transformation (%)
Alsoghier 2023	140	47 ± 13	4:3	Not stated	Diabetes common (40% ≥ 1 condition)	Not stated	Buccal mucosa (52%)	Not stated	Not stated	12% dysplasia
Alhelo 2020	50	48	2.5:1	N/A	Multiple incl. DM (30%), HTN, HCV	Reticular (98%), erythematous (66%), ulcerative	Buccal (90%), tongue (50%), gingiva, lips, palate	Topical corticosteroids, systemic + topical, reassurance	68%	NR
Alblowi and Binmadi 2018	4/1248	47.5 ± 13.5	1:1	0.3%	NR	NR	NR	NR	NR	NR
Al Wayli 2016	42	24–63	Female only	0.8%	NR	Reticular most common	Buccal mucosa	NR	NR	NR
Halawani 2013	47	49 ± 12.8	39:8	44.7% OLP in LP	HCV infection	NR	NR	NR	NR	NR
AlMobeeriek and Aldosari 2009	9	38.2	1.36:1	0.3%	NR	Reticular (98%), erosive (66%), plaquelike (8%)	Buccal (80%), tongue (38%)	NR	NR	NR
Ali and Suresh 2007	80	43.4	1:3	NR	DM: 35% study vs 15% control	Reticular, erosive, atrophic, vesicular, multiple	Buccal most common	NR	NR	NR
Tonsi and Samdani 2005	12/114	40.7	0.9:1	10.5%	HCV 10.5% vs 4.6% control	NR	NR	NR	NR	NR
ElRifaei 1999	34	NR	NR	NR	NR	NR	NR	NR	NR	NR
Salem 1989	72/4277	49	0.8:1	1.7%	DM (30%), heart disease, CKD, HBV	Reticular, plaque, atrophic, erosive	Buccal (86%), gingiva, tongue, palate, lips	Topical steroid + anaesthetic	NR	5.6%
Mani 1985	4/674	NR	0.76:1	0.6%	None	NR	NR	NR	NR	0.2%
NR = not reported; DM = diabetes mellitus; HTN = hypertension; HBV/HCV = hepatitis B/C virus.

## RESULTS

### Study Selection and Characteristics

The systematic search initially identified 126 records through a comprehensive search of multiple electronic databases including PubMed, Scopus, and the Saudi Digital Library. After removal of duplicates and application of inclusion and exclusion criteria, 11 studies met the eligibility criteria for detailed analysis (Fig 1). These comprised eight cross-sectional studies, predominantly retrospective in nature, and three case-control studies. The included studies spanned a wide period from 1985 to 2023 and were conducted across multiple regions of Saudi Arabia, including Riyadh, Jeddah, Makkah, Dammam, and Jizan, reflecting a reasonable geographical diversity, albeit with an urban and tertiary care center bias (Table 3).

**Table 3:** Characteristics of oral lichen planus studies in Saudi Arabia

Author (year)	Study aim	Study design	Location	Period	Sample size	Sampling method
Alsoghier et al (2023)	Assess demographics and clinicohistological characteristics of OLP and dysplasia relevance	Cross-sectional retrospective	King Saud University, Riyadh	Sept. 2022 – Sept. 2023	140	Convenience sampling of available records
Alhelo et al (2020)	Describe epidemiology and clinicopathology of OLP	Cross-sectional retrospective	King Abdulaziz University Dental Hospital (KAUDH), Jeddah	June 2012 – June 2018	50 OLP patients from 10,000 dental patients	Convenience sampling from oral medicine referrals
Alblowi and Binmadi (2018)	Frequency and distribution of gingival lesions biopsied (19962016)	Cross-sectional retrospective	KAUDH Oral Pathology Lab, Jeddah	1996 – 2016	1,248 oralmaxillofacial lesions (119 gingival)	Possibly convenience sampling of all biopsy submissions
Al Wayli et al. (2016)	Prevalence and distribution of oral mucosal lesions among Saudi female patients	Cross-sectional	AlYamamah Hospital, Riyadh	2005 – 2010	5,543 females (379 lesions; 42 OLP)	Consecutive clinical attendees
Halawani (2013)	Prevalence of anti-HCV antibodies and genotypes in LP patients	Case–control	King Khalid University Hospital, Riyadh	2008 – 2010	47 (45 Saudis)	Convenience sampling of LP patients and controls
AlMobeeriek and Aldosari (2009)	Type and extent of oral lesions	Cross-sectional	College of Dentistry, Riyadh	~2006 – 2009	2,552 outpatients (383 lesions; 9 OLP)	Consecutive attendees
Ali and Suresh (2007)	Relationship between OLP, transaminase, and HCV	Case–control	King Faisal University, Dammam	2006 – 2007 (est.)	80 confirmed OLP	Not specified
Tonsi and Samdani (2005)	AntiHCV antibodies and clinical LP	Cross-sectional	Alawi Tonsi Hospital, Makkah	1999 – 2001	114 LP patients (12 OLP)	Not specified
ElRifaei (1999)	HBV/HCV and aminotransferase in OLP	Cross-sectional	Dammam Central Hospital	Not specified	34 OLP patients; 32 controls	Convenience sampling
Salem (1989)	OLP prevalence	Cross-sectional	King Fahd Central Hospital, Jizan	1982 – 1987	4,277 patients (72 OLP)	Likely consecutive attendees
Mani (1985)	Oral cancer and precancerous lesion prevalence	Cross-sectional	King Saud University, Riyadh	Jan. – Dec. 1983	674 patients	Consecutive first visits


Sample sizes of the studies varied substantially. The largest cohort included more than 5500 participants,^14^ mainly female, whereas the smallest case series reported 34 patients.^22^ Several studies sourced data from dental or dermatology clinics, with sampling predominantly based on convenience or consecutive patient attendance rather than random sampling.^3,5,10,13,32,40,42^ This introduces potential bias but reflects real-world clinical populations in Saudi Arabia.

### Quality Assessment and Risk of Bias

The methodological quality of included studies was appraised using Joanna Briggs Institute checklists, tailored to study design differences (Tables 1 and 2). Most cross-sectional studies demonstrated moderate to high methodological rigor, with scores ranging from 5.5 to 7.7 out of 8. However, one older study^32^ scored significantly lower (2.5), indicating considerable risk of bias related to unclear inclusion criteria and inadequately described exposure measurements. Common limitations across the newer studies included unclear handling of confounding variables and insufficient description of participant recruitment, which may affect internal validity.

The two case-control studies^6,26^ were similarly robust, each scoring 9 out of 10, with minor concerns regarding confounding control strategies (Table 2). Risk-of-bias assessments visualized in traffic-light plots and summary figures further corroborated these findings, highlighting overall acceptable quality but identifying particular weaknesses in confounder mitigation and participant description (Figs 2 to 5).

### Prevalence and Demographic Features of OLP

The prevalence of oral lichen planus reported in Saudi Arabia exhibited notable heterogeneity, ranging from as low as 0.3% in studies involving broad dental patient populations^3,10^ to as high as 11.1% within cohorts selectively enriched for lichen planus or hepatitis C virus (HCV) infection risk^26,42^ (Table 4). This disparity is linked in part to differences in study population characteristics, sampling methodologies, and diagnostic criteria—some studies relied purely on clinical diagnosis while others integrated histopathological confirmation.^3,5,6,10,13,14,22,26,32,40,42^

Age distributions across cohorts averaged in mid-to-late middle adulthood (approximately 38 to 49 years), consistent with international epidemiological patterns of OLP. Sex ratios mostly favored females, with female:male ratios generally ranging from 1.3:1 to 2.5:1, confirming the known higher incidence of OLP in women, although exceptions existed in HCV-focused studies,^5,26,42^ which showed a slight male predominance (Table 4).

### Clinical Presentation and Diagnostic Approaches

Clinical features of OLP reported in these studies largely align with global observations. The reticular form was consistently the most common subtype, observed in up to 98% of cases,^5,10^ followed by erythematous and erosive variants. Lesion distribution predominantly involved the buccal mucosa, with secondary sites including the tongue, gingiva, lips, and palate—a pattern consistent with OLP’s typical anatomical predilection (Table 4).

Diagnostic approaches differed across studies. Several incorporated histopathological assessments to confirm OLP diagnoses and exclude mimicking conditions such as lichenoid drug reactions or autoimmune blistering disorders.^3,5^ Others relied on clinical criteria alone, which may increase diagnostic uncertainty. Notably, the application of contemporary diagnostic guidelines such as those from the American Academy of Oral and Maxillofacial Pathology was limited, although newer studies increasingly emphasized combined clinical and microscopic diagnosis for accuracy (Table 4).^13^ Notable symptoms were frequently pain or burning sensations, with symptomatic cases representing an estimated 54% to 68% of patients in some cohorts,^5^ underscoring the clinical relevance and impact on quality of life.

### Associated Systemic Conditions

A wide range of systemic comorbidities were reported among OLP patients, with diabetes mellitus and hypertension being most common. Several studies also detailed a notable prevalence of thyroid disorders and hyperlipidemia among participants.^5,40^ Importantly, hepatitis C virus infection appeared frequently associated with OLP in Saudi cohorts. The seroprevalence of HCV among OLP patients in included case-control studies ranged from 10.5% to 44.7%, markedly higher than the national average prevalence of about 0.8%^6,26^ (Table 4). These findings highlight a probable etiological or associative link warranting routine HCV screening in OLP patients, especially in regions with higher endemicity. Other environmental or behavioral risk factors such as tobacco use, local dietary habits, or exposure to allergens and dental materials were insufficiently explored, marking an area for further investigation.

### Management and Response to Treatment

Treatment modalities for OLP across Saudi studies varied. The most frequent therapeutic approach involved topical corticosteroids, employed either alone or in combination with systemic steroids or other immunomodulatory agents.^5,40^ Supportive measures, including patient education and reassurance, were also common.

Clinical response rates to treatments were generally moderate. For instance, Alhelo et al^5^ observed clinical improvement in approximately 68% of treated patients, while a substantial proportion remained refractory or experienced no significant symptom relief. However, patient adherence and follow-up rates were often inadequate, with up to 34% of patients refusing or discontinuing treatment, reflecting gaps in patient engagement or potential lack of standardized care pathways.

### Malignant Transformation and Dysplasia Prevalence

Malignant transformation rates of OLP among Saudi patients fell within the expected global range but exhibited variability, reported between 0.2% and 5.6%.^32,40^ Alsoghier et al^13^ reported the presence of epithelial dysplasia in up to 12% of cases based on histopathological assessment. These figures reinforce that OLP, particularly erosive and atrophic subtypes, carry a non-negligible risk of progression to oral squamous cell carcinoma.

Follow-up durations necessary to capture malignant progression were generally limited or absent in most studies, impeding robust risk stratification. Salem^40^ uniquely provided longitudinal data with a mean follow-up of approximately 3.5 years. The influence of confounders, notably tobacco use, systemic health status, and treatment regimens, was insufficiently adjusted for, again limiting conclusions regarding independent malignant risk.

### Subgroup and Comparative Analyses

Analysis stratified by diagnostic methodology revealed that studies incorporating histopathological confirmation tended to report lower OLP prevalence rates (0.3% to 5.6%) relative to those relying solely on clinical diagnosis (up to 11.1%). This discrepancy reinforces the importance of standardized diagnosis for epidemiological precision. Geographically, higher prevalence and stronger HCV associations were observed in western regions (Jeddah, Makkah), areas known for higher hepatitis C endemicity, compared to relatively lower rates in central regions (Riyadh) (Table 4).

## DISCUSSION

The present systematic narrative review synthesizes evidence from 11 clinical studies^3,5,6,10,13,14,22,26,32,40,42^ examining oral lichen planus (OLP) within the Saudi Arabian population. Three key findings emerge prominently: the wide variability in reported prevalence rates, a notable association between OLP and hepatitis C virus (HCV) infection, and the documented rates of malignant transformation.

### Prevalence Variability and Diagnostic Challenges

The reported prevalence of OLP in Saudi Arabia spans a striking range—from as low as 0.3% to as high as 11.08%,^3,5,6,10,13,14,22,26,32,40,42^ which is considerably broader than global estimates, typically ranging from 1% to 1.5%.^24^ Such heterogeneity likely stems from several intertwined factors. First, variation in diagnostic criteria creates substantial inconsistency; some studies rely solely on clinical diagnosis, while others incorporate histopathological confirmation.^3,5,6,10,13,14,22,26,32,40,42^ Histopathologically confirmed cases tend to report lower prevalence compared to clinical-only diagnoses, potentially reflecting over-diagnosis or misclassification when strict tissue-based criteria are not applied.^18^ Second, study settings differ—population-based screening is limited, with most data derived from hospital-based or specialty clinic cohorts, often leading to selection bias toward symptomatic or referred cases.^13,40^ Third, geographic disparities within Saudi Arabia, emerging from distinct environmental exposures or genetic variations, may influence disease distribution; Western regions like Jeddah and Makkah show higher reported prevalence, possibly correlated with increased exposure to HCV or other region-specific risk factors.^8,9,26^ These findings underscore the urgent need for standardized diagnostic protocols adopting the internationally recognized histopathological and clinical criteria, aiming to unify case definitions and enable comparability across future Saudi studies.

### Association of OLP and Hepatitis C Virus

A salient and recurrent theme in the reviewed literature is the elevated seroprevalence of HCV among OLP patients in Saudi studies relative to the general population’s baseline HCV rate.^4^ Specifically, seropositivity in OLP cohorts varies between 10.5% and 44.7%, significantly exceeding population norms.^5,26,42^ Mechanistically, HCV infection may provoke aberrant immune responses, including molecular mimicry or chronic antigenic stimulation, leading to the persistent T-cell mediated epithelial injury characteristic of OLP.^31^ The implications for clinical practice in Saudi Arabia are profound: routine HCV screening for patients presenting with OLP could facilitate early identification of viral comorbidity and allow tailored antiviral therapies that may influence OLP course and malignant potential. Nevertheless, the predominance of data from single-center studies cautions against overgeneralization; multicenter, prospective investigations are warranted to validate this association and unravel its clinical significance within diverse Saudi subpopulations. This also aligns with the global discourse identifying regional variation in the OLP-HCV nexus.^44^

### Malignant Transformation Potential

Malignant transformation of OLP into oral squamous cell carcinoma remains a clinical concern, with rates globally reported between 0.4% and 5%.^23,44^ Within Saudi studies, observed transformation rates ranged from 0.2% to 5.6%, consistent with global figures but highlighting the necessity for cautious surveillance.^28,32,40,43^ Importantly, these rates derive primarily from retrospective studies with limited longitudinal follow-up, typically averaging around 3–5 years, a timeframe potentially insufficient to capture all cases of malignant progression.^40^ Furthermore, certain clinical subtypes—especially erosive and atrophic forms—demonstrate a predilection for dysplastic changes and transformation, though subtype-specific risk stratification remains underexplored in Saudi cohorts.^8,13^ Another complicating factor is the relative paucity of adjustment for confounding exposures such as tobacco and alcohol use, which independently increase oral cancer risk and may synergistically elevate transformation likelihood in OLP patients.^8,10, 15,35,37^ This insufficiency necessitates augmented prospective monitoring frameworks incorporating clinical, histopathological, and molecular parameters to refine risk prediction models and optimize patient management pathways.

### Therapeutic Landscape and Patient Management

The therapeutic approaches across Saudi studies reveal a predominant reliance on topical corticosteroids, which remain the mainstay for symptom control in OLP.^5,41^ Nevertheless, variations in treatment regimens and patient adherence patterns introduce complexity to efficacy assessments. For instance, nearly half of the patients either do not commence treatment or discontinue follow-up, indicating gaps in patient education, motivation, or access.^5^ Notably, psychological comorbidities such as anxiety and depression, recognized internationally as exacerbating factors for pain perception and quality of life in OLP, remain under-addressed in Saudi clinical settings despite their likely relevance.^1^ Optimizing patient counseling, integrating psychosocial support, and standardized treatment protocols—including possibly antiviral therapy in HCV-positive cases—could improve adherence and clinical outcomes.^1^ A proposed management algorithm prioritizing initial HCV screening, histological diagnosis, corticosteroid therapy, structured follow-ups every 3–6 months for high-risk patients, and specialist referrals for dysplastic lesions or refractory disease is timely and warranted.

### Study Strengths, Limitations, and Future Directions

This review represents the first comprehensive synthesis of OLP data specific to Saudi Arabia and benefits from rigorous methodological critique using standardized risk of bias tools. The inclusion of both cross-sectional and case-control studies provides a balanced perspective on prevalence, risk factors, and outcomes.^3,5,6,10,13,14,22,26,32,40,42^ Nevertheless, limitations permeate. The substantial heterogeneity across regions, diagnostic criteria, and study designs precluded formal meta-analysis and accentuated interpretative challenges. Additionally, rural and underserved populations remain largely unstudied, limiting generalizability. Finally, a lack of prospective longitudinal data restricts insight into disease progression and malignant transformation trajectories.

Future research should prioritize well-designed multicenter cohort studies employing validated diagnostic criteria and incorporating comprehensive assessments of systemic comorbidities, lifestyle exposures, and psychosocial factors. Molecular profiling, including cytokine expression, genetic polymorphisms, and viral-host interactions, holds promise for elucidating pathogenesis and refining personalized risk stratification. Additionally, the development of a national registry for OLP patients would facilitate robust surveillance, early detection of malignancy, and improved care coordination. Patient-reported outcomes and quality-of-life metrics should also be integrated into research and clinical practice to holistically address the burden of OLP within Saudi Arabia.

## CONCLUSION

OLP in Saudi Arabia demonstrates considerable epidemiological diversity and is associated with significant comorbidity, including an elevated prevalence of HCV infection and a comparable risk of malignant transformation to global reports. The findings highlight the imperative for harmonized diagnostic criteria, routine HCV screening, and enhanced patient management frameworks. Moving forward, multicenter prospective research with longitudinal follow-up and integrated molecular and psychosocial assessments will be essential to optimize clinical outcomes and inform public health strategies targeting this chronic, potentially malignant oral condition within the Saudi context.
